# Supplementary material for: Condition dependent strategies of egg size variation in the Common Eider Somateria mollissima
Source: PLoS One. 2020 Jul 27;15(7):e0226532. doi: 10.1371/journal.pone.0226532 (PMC7384649; doi:10.1371/journal.pone.0226532)
Supplement: S1 File — (DOCX) [file pone.0226532.s001.docx]

**Supporting information**

**S1 Analyses:**

**Test for the effect of clutch size on w_hatch_**

To investigate whether clutch size differences might have affected the estimates of of W_start_, we tested how W_hatch_ related to clutch size and tarsus^3^ for females with clutch sizes of 3 to 5 eggs where hatching (ducklings or piping/cracking eggs) was recorded (N = 150). We used a mixed model with clutch size, tarsus^3^ and their interaction as fixed variables and year as random variable. Tarsus was included as the cubic root to scale the length measure to a spatial dimension that reflected weight. Clutch size and the interaction clutch size * tarsus^3^ did not affect W_hatch_ (Mixed model, clutch size: F_2, 137_ = 0.23, p = 0.794; clutch size * tarsus^3^: F_2, 137_ = 0.16, p = 0.852), whereas tarsus^3^ showed a significant positive relation (F_1, 137_ = 11.1, p = 0.001, slope=0.0013). This analysis included an additional 112 females caught with ducklings in the nest, which were not part of the egg-size sample.
