# Supplementary material for: Condition dependent strategies of egg size variation in the Common Eider Somateria mollissima
Source: PLoS One. 2020 Jul 27;15(7):e0226532. doi: 10.1371/journal.pone.0226532 (PMC7384649; doi:10.1371/journal.pone.0226532)
Supplement: S1 Table — (DOCX) [file pone.0226532.s002.docx]

**Supporting information**

**S2 Table. Distribution of clutch sizes.**

| Clutch size | Number of clutches |
| --- | --- |
| **1** | 14 |
| **2** | 57 |
| **3** | 185 |
| **4** | 395 |
| **5** | 415 |
| **6** | 33 |
| **7** | 2 |
