# Supplementary material for: Condition dependent strategies of egg size variation in the Common Eider Somateria mollissima
Source: PLoS One. 2020 Jul 27;15(7):e0226532. doi: 10.1371/journal.pone.0226532 (PMC7384649; doi:10.1371/journal.pone.0226532)
Supplement: S2 Table — (DOCX) [file pone.0226532.s003.docx]

**Supporting information**

**S3 Table. Least square mean estimates (LS means) in mean clutch size for year and test of post hoc pairwise differences.**

| LS means | | 1993 | 1994 | 1995 | 1996 | 1997 | 1998 | 1999 | 2000 |
| --- | --- | --- | --- | --- | --- | --- | --- | --- | --- |
| 1993 | 4.300 |  | 0.114 | 0.447 | 0.971 | **0.017** | **0.050** | 0.083 | 0.572 |
| 1994 | 4.080 |  |  | 0.323 | 0.071 | 0.359 | 0.700 | 0.920 | 0.275 |
| 1995 | 4.196 |  |  |  | 0.395 | 0.052 | 0.153 | 0.250 | 0.860 |
| 1996 | 4.295 |  |  |  |  | **0.006** | **0.023** | **0.044** | 0.542 |
| 1997 | 3.973 |  |  |  |  |  | 0.570 | 0.384 | **0.049** |
| 1998 | 4.036 |  |  |  |  |  |  | 0.761 | 0.134 |
| 1999 | 4.069 |  |  |  |  |  |  |  | 0.214 |
| 2000 | 4.218 |  |  |  |  |  |  |  |  |
