# Supplementary material for: Condition dependent strategies of egg size variation in the Common Eider Somateria mollissima
Source: PLoS One. 2020 Jul 27;15(7):e0226532. doi: 10.1371/journal.pone.0226532 (PMC7384649; doi:10.1371/journal.pone.0226532)
Supplement: S3 Table — Post hoc pairwise tests were estimated with least square mean. (DOCX) [file pone.0226532.s004.docx]

**Supporting information**

**S4 Table. Test of pairwise differences in egg volume between clutch sizes (first to third egg). Post hoc pairwise tests were estimated with least square mean.**

|  | Clutch size | Clutch size | Estimate | SE | DF | t | p |
| --- | --- | --- | --- | --- | --- | --- | --- |
| First egg | 1 | 2 | -5.094 | 2.665 | 804 | -1.91 | 0.056 |
| First egg | 1 | 3 | -7.715 | 2.507 | 804 | -3.08 | **0.002** |
| First egg | 1 | 4 | -9.171 | 2.465 | 804 | -3.71 | **<0.001** |
| First egg | 1 | 5 | -7.424 | 2.469 | 804 | -3.01 | **0.003** |
| First egg | 1 | 6 | -7.688 | 2.853 | 804 | -2.69 | **0.007** |
| First egg | 2 | 3 | -2.621 | 1.246 | 804 | -2.1 | **0.036** |
| First egg | 2 | 4 | -4.077 | 1.169 | 804 | -3.49 | **<0.001** |
| First egg | 2 | 5 | -2.330 | 1.167 | 804 | -2 | **0.046** |
| First egg | 2 | 6 | -2.594 | 1.845 | 804 | -1.41 | 0.160 |
| First egg | 3 | 4 | -1.457 | 0.741 | 804 | -1.96 | **0.050** |
| First egg | 3 | 5 | 0.291 | 0.738 | 804 | 0.39 | 0.694 |
| First egg | 3 | 6 | 0.027 | 1.609 | 804 | 0.02 | 0.987 |
| First egg | 4 | 5 | 1.75 | 0.600 | 804 | 2.91 | **0.004** |
| First egg | 4 | 6 | 1.484 | 1.551 | 804 | 0.96 | 0.339 |
| First egg | 5 | 6 | -0.263 | 1.548 | 804 | -0.17 | 0.865 |
| Second egg | 2 | 3 | -4.442 | 1.303 | 796 | -3.41 | **0.007** |
| Second egg | 2 | 4 | -6.803 | 1.222 | 796 | -5.57 | **<.0001** |
| Second egg | 2 | 5 | -8.015 | 1.220 | 796 | -6.57 | **<.0001** |
| Second egg | 2 | 6 | -6.689 | 1.929 | 796 | -3.47 | **<0.001** |
| Second egg | 3 | 4 | -2.361 | 0.775 | 796 | -3.05 | **0.002** |
| Second egg | 3 | 5 | -3.574 | 0.771 | 796 | -4.63 | **<.0001** |
| Second egg | 3 | 6 | -2.25 | 1.682 | 796 | -1.34 | 0.182 |
| Second egg | 4 | 5 | -1.212 | 0.627 | 796 | -1.93 | 0.054 |
| Second egg | 4 | 6 | 0.114 | 1.621 | 796 | 0.07 | 0.945 |
| Second egg | 5 | 6 | 1.327 | 1.618 | 796 | 0.82 | 0.413 |
| Third egg | 3 | 4 | -2.756 | 0.720 | 752 | -3.83 | **<0.001** |
| Third egg | 3 | 5 | -4.791 | 0.717 | 752 | -6.69 | **<.0001** |
| Third egg | 3 | 6 | -6.690 | 1.562 | 752 | -4.28 | **<.0001** |
| Third egg | 4 | 5 | -2.035 | 0.582 | 752 | -3.5 | **<0.001** |
| Third egg | 4 | 6 | -3.932 | 1.505 | 752 | -2.61 | **0.009** |
| Third egg | 5 | 6 | -1.897 | 1.503 | 752 | -1.26 | 0.208 |
