# Supplementary material for: Condition dependent strategies of egg size variation in the Common Eider Somateria mollissima
Source: PLoS One. 2020 Jul 27;15(7):e0226532. doi: 10.1371/journal.pone.0226532 (PMC7384649; doi:10.1371/journal.pone.0226532)
Supplement: Attachment — Submitted filename: Response to Reviewers2.docx [file pone.0226532.s005.docx]

**PLOS ONE**

Online re-submission PONE-D-19-33049

*Review response*

Dear Editor!

**With responses to ADDITIONAL NEW EDITORIAL requests/comments in yellow capital letters.**

On behalf of my co-author, Thorsten J. S. Balsby and I, I hereby submit the revised research paper entitled “**Condition dependent strategies of egg size variation in the Common Eider Somateria mollissima**”.

Please below in **red/bold** find a detailed response to all the excellent review comments and changes addressed. We have of course used all these excellent suggestions. All changes are shown in the highlighted version of our revised manuscript.

I am looking forward hearing from you.

Sincerely yours,

*Thomas Kjær Christensen
Senior researcher, PhD*
Institut for Bioscience, Aarhus Universitet
Grenåvej 14, 8410 Rønde, Danmark
Mail: [tk@bios.au.dk](mailto:tk@bios.au.dk) | tlf.: +45 87158854  cell: +45 40256813

Response to editors and reviewers

Editors/Journal requirements

1. Please ensure that your manuscript meets PLOS ONE's style requirements, including those for file naming. The PLOS ONE style templates can be found at <http://www.plosone.org/attachments/PLOSOne_formatting_sample_main_body.pdf> and <http://www.plosone.org/attachments/PLOSOne_formatting_sample_title_authors_affiliations.pdf>. **We believe that we have followed all instructions for manuscript style requirements outlined for PLOS ONE manuscript (the link provided does, however, not work!)**

2. In your Methods section, please provide additional information regarding the permits you obtained for the work. Please ensure you have included the full name of the authority that approved the field site access and, if no permits were required, a brief statement explaining why. **We have now included the information on landowners that provided access to the field area.**

3. We note that you have indicated that data from this study are available upon request. PLOS only allows data to be available upon request if there are legal or ethical restrictions on sharing data publicly. For more information on unacceptable data access restrictions, please see <http://journals.plos.org/plosone/s/data-availability#loc-unacceptable-data-access-restrictions>.

In your revised cover letter, please address the following prompts:

a) If there are ethical or legal restrictions on sharing a de-identified data set, please explain them in detail (e.g., data contain potentially sensitive information, data are owned by a third-party organization, etc.) and who has imposed them (e.g., an ethics committee). Please also provide contact information for a data access committee, ethics committee, or other institutional body to which data requests may be sent. **The data does not contain potentially sensitive information, nor represents any ethical problems. Data is owned by a third-party, A/S Øresundsbrokonsortiet, which approved the use of the data for this study. Data was collected as a part of a comprehensive Impact Assessment study commissioned by the A/S Øresundsbrokonsortiet, and is available for researchers upon request and acceptance from the A/S Øresundsbrokonsortiet (contact via kontakt@oresundsbron.com).**

b) If there are no restrictions, please upload the minimal anonymized data set necessary to replicate your study findings as either Supporting Information files or to a stable, public repository and provide us with the relevant URLs, DOIs, or accession numbers. For a list of acceptable repositories, please see <http://journals.plos.org/plosone/s/data-availability#loc-recommended-repositories>. **Please see comments under 3a) above.**

**We confirm that the authors of this study had no special access privileges to the data others would not have.**

We will update your Data Availability statement on your behalf to reflect the information you provide.

4. Please include captions for your Supporting Information files at the end of your manuscript, and update any in-text citations to match accordingly. Please see our Supporting Information guidelines for more information: <http://journals.plos.org/plosone/s/supporting-information>. **Captions for Supporting Information files have been included.**

5. Please upload a copy of Figure 7 which you refer to in your text on page 16. Or if the figure is no longer to be included as part of the submission please remove all reference to it within the text. **THE FIGURE 7 REFERED TO IN THE TEXT AT PAGE 16 IS A FIGURE IN REFERENCE 7 AS STATED. IF IT CAUSE CONFUSION TO REFER TO A FIGURE IN A REFERENCE BY WRITING: “Figure 7 in [7]” and “Table 1 in [9]”, WE WILL OF CAUSE CHANGE THIS TO “[7;9;see also 10]”.**

Reviewer #1:

This article tests if common eider females adaptively adjust within-clutch egg size depending on clutch size, laying order and their own body condition. The authors use long-term, longitudinal data on a population of common eiders to analyse how variation in clutch size depends on maternal body condition; and how variation in egg size depends on clutch size and laying order, at the population and the individual levels.

**Comments to the following paragraph is separated into 6 answers that are presented below the paragraph. Answer reference numbers are inserted into the reviewer text.**

The study has potential as the results are of interest, and are based on a strong dataset. The analyses at both the population and the individual levels bring very important information on egg size variation patterns. **1):** The study fails, however, to address the hypothesis the authors wish to test: that variation in within-clutch egg size patterns is an adaptive maternal adjustment. **2):** The authors analyse the direct relationship between maternal condition and clutch size, and between clutch size, laying sequence and egg size, but do not test the effect of within-clutch egg size variation patterns on any fitness trait (reproductive success in terms of number of hatchlings or offspring survival, or survival of the first egg). They instead rely on literature to make that link. Although literature on birds does state that egg size is related to maternal reproductive success, it has not, from the references listed, been conclusively tested in this population or species **3):** and is not tested in this article. **4):** Moreover, laying sequence has been found to be as strong a predictor of hatching success and hatchling survival as egg size, according to some references cited in the manuscript (e.g. ref. 11). **5):** While it seems fair to interpret and discuss the results of this study in the larger context of reproductive tactics and adaptive adjustment framework, the aim and hypothesis should be reformulated and nuanced to focus more on and better fit with what the analyses actually test. **6):** If data are available to investigate the adaptive value of within-clutch variation (for example by testing survival probability of first eggs in 4- and 5-egg clutches), that should be done.

**Answer 1: We appreciate the reviewers point. We have now emphasized what we test in this study and we think that these changes have an adaptive value. We acknowledge that we do not test the adaptive value explicitly, but the changes and variation in egg sized are consistent with an adaptive interpretation. However, we actually show that females adapt within-clutch egg size in relation to their body condition and that this adaptation is linked to a change in clutch size. From the other comments of reviewer #1 we find that the interpretation of missing the aim of the study relates to not presenting data on the ultimate fitness, namely hatchling/duckling survival from eggs of difference size. It is true that we do not present data on these topics, because we do not have data on these topics. They are, however well documented in the literature
Answer 2: It’s correct that we do not test the effect of egg-size on reproductive success or offspring survival. However, there are a few relevant references, which address these topics. Not many studies have tested the relation between egg size and fitness, but the link is shown for eiders from egg size to duckling growth until 12 days after hatching (ref 9) and survival of duckling of different size to recruitment as breeders (ref 26). We therefore disagree with the statement that such a relation between investment and potential fitness consequences has not been documented for the eider.
Answer 3: No, not intended, as we do not have data on this!
Answer 4: We do not exclude a relation between egg survival and laying sequence. We actually think that our results support this hypothesis and certainly do not exclude these findings!
Answer 5: The hypothesis has been reformulated to account for the reviewers points. By taking in the comments from the reviewers, we find that we have sharpened the formulations so the aim and results are now precisely described.
Answer 6: This is not a study on individual survival, so we cannot conduct such analysis.**

The methods section needs more work. The data collection part is sufficiently detailed, but the statistics part requires attention. Some of the results presented (clutch size, L202-206 and L216-218) are from analyses that are not described in the methods. Generally, some paragraphs are unclear and need reformulation or more details (e.g. L140-143, or the absence of details concerning linear model selection methods, linear model conformation to assumptions when applicable (homoscedasticity, normality)).

**Answer: Good point; we should have mentioned these tests and addressed the assumptions. The descriptions of these analyses have now been included in the statistics paragraph. At the end of this paragraph, we have also mentioned that residual did not deviate from the assumptions for these tests.**

The structure of the manuscript is also at some place surprising, and overall inconsistent. The results are presented in a different order from that in the methods. The same goes for the discussion. The methods section describes an analysis of variation in first, second and third egg sizes across different sizes of clutch (L172-173) in very little details, an analysis of within-clutch egg size variation in relation to laying sequence and clutch size (L174-182), a third analysis of within-individual clutch size variation (L183-191), and a last analysis of within-clutch egg size variation for females with repeated measurements that changed clutch size (L191-194). The results section first presents descriptive results about clutch size and results from a linear model that seem to test the effect of year and female condition on clutch size, but that is not described in the methods section (L201-218). The section then presents descriptive data and results from the analysis body mass and clutch size variation between years for females captured several times (L221-232). Then are presented descriptive data on egg size (L235-239) and results from the linear model testing variation in egg size within clutches, in relation to laying order (L240-271). Finally, the authors present results of within-clutch egg variation according to clutch size for females that changed clutch size in subsequent years (L275-296).

**Answer: Ok this is a fair point. We have rearranged the statistics paragraph, so the order of the tests now match the order of presentation in the results and the discussion. To ease the navigation between the statistics paragraph and the results section we have inserted the sub-headlines from the results section in the statistics section. We now find that there is a consistent structure between methods, results and discussion.**

There is also a lack of consistency in terminology (to distinguish tactic from strategy, see Gross 1996, in TREE; egg size and egg volume are used inter-changeably, as are body mass and body condition) and in the way the results are presented (sometimes F values, sometimes effect sizes but referred to as “slope” (L218) or “estimate” (L230), sometimes reporting the R² value of the model (L205)). It would be more informative to present the effect of the variables tested with linear models by giving the effect size (β) and standard error (or any error assessment) rather than F values.

**Answer: We report R^2^ whenever possible, but R^2^ is not always an option, such as in mixed models in SAS. We report slopes for variables where this is possible, except for tables where slopes would make tables too big. We do not agree on the suggested practice of reporting slope/effect sizes and SE instead of F-values. F-values forms the basis for standardized comparison, whereas slopes depend on the numeric values of the observations. Furthermore, most readers will readily understand F-values, but not just slope and SE.**

Finally, there are throughout the manuscript several spelling, grammar and punctuation mistakes that need attention.

**Answer: We have corrected grammar to our best ability.**


Detailed comments (with line numbers) (Answers have been inserted in brackets at each point)

L22-23: the sentence needs to be reformulated: the authors looked at the effect of female body condition on clutch size, and then at the effect of laying sequence and clutch size on egg size, but did not analyse the direct relationship between female body condition and egg size. **(Some corrections have been made, so readers do not get the impression that we analyse the direct relationship between female condition and egg size.)**

L27-28: please reformulate, as “data from recaptured females…” only refers to the individual level of the analyses **(changes made to separate population and individual level)**

L61: please remove the commas: “Alternative but not mutually exclusive hypotheses have related…” **(done)**

L66: “have proved to fully explain the observed…” **(done)**

L74-75: what about literature that found a trade-off between size and number of offspring? (even in Anseriformes e.g. Christians 2000 Trade-offs between egg size and number in waterfowl… or Figuerola and Green 2006 A comparative study of egg mass and clutch size in the Anseriformes) **(The two studies referred to address the egg-size/clutch size trade-offs between species and not within species. We have added the word “intraspecific” in the text so that readers are focused on “..adaptive intraspecific changes…” that may occur within individuals, and which are only circumstantially documented)**

L77-80: sentence unclear, needs reformulation **(changes have been made to clarify the sentence: inserting “egg laying” instead of “breeding” is more precise)**

L86: no need for a comma: “…females is mainly adjusted…” **(comma deleted)**

L86-88: these studies do not show that larger clutches, laid by females in good condition, have a higher reproductive success than small clutches **(Ankney & MacInnes (ref 14), Erikstad et al. (ref 23) and Sedinger (ref 39) all describe or argue for this relationship. Deschamps et al. (ref 37) is less direct, but mention the same relation)**

L89: “records of egg size” **(done)**

L89-93: are these not results from the analyses described in the paper? It seems so, thus it is surprising to find them presented in the introduction. **(Agreed that this is a slightly alternative way of introducing the central subject of the present analyses. However, this information is essential for readers to understand the hypotheses, which we test in the paper. We do, therefore, not find that this introduction disqualifies the manuscript setup, but rather focus the readers on the relevant question and key point of this paper.)**

L93: please specify the direction of the difference. **(If we specify the direction, then we really would present results here. For this reason we do not present the direction of the egg size change)**

L94-96: please provide references **(references inserted at this point)**

L99: “depending on clutch size” **(done)**

L100: should be reformulated: the paper analyses the effect of body condition on clutch size, but body condition is not included in the main analysis of the effect of clutch size and laying sequence on egg size. **(Changes made)**

L108: it seems that all data were collected between 1993 and 2000, so it does not seem relevant to specify “Data on clutch size and egg size” **(done)**

L109-110: it sounds weird to describe past density in a present tense; this sentence should be reformulated to indicate it was the density estimated at the time the data was collected. **(changes made)**

L127: verb missing: “tarsus length […] were recorded” **(done)**

L135: it is not very clear what “related to the laying sequence” means (numbered? ordered?), this part should be reformulated **(This part has been reformulated)**

L138: the fact that egg size in the MS will refer to egg volume should be introduced here, not L235. **(changes made)**

L139 and throughout: please be consistent between body mass and body condition **(We have been very aware and consequent in the use of the term “body mass” and “body condition”. We only use the term “body mass” in description of measured body mass of females. In the analyses of recaptured females we also use the term body mass, as this relates to the same individual females. The term “body condition” is used strictly in population-related analyses when relating measured body mass to measured structural size (tarsus length). We originally defined body condition as “mass corrected for structural size in line 139, but changes have been made (see next comments)**

L139-143: this is unclear, please reformulate **(changes have been made to make this section more simple and understandable. This has been done by rephrasing and restructuring the text.)**

L149-150: it is not clear if body mass refers to actual measured mass or the calculation of body condition **(Corrections** **have been made to clarify this)**

L150: it is unclear if “annual body mass at hatching” refers to maternal or hatchlings body mass **(now specified that it is female body mass)**

L155-164: this paragraph should be moved to supplementary, and referred to in the paragraph detailing the calculation of female body condition in terms of how the potential effect of such and such variables on body condition was considered and controlled for. Moreover, details are missing concerning the structure of the model ran and the model selection process: it is not clear if the values presented are from a single model. If so, F values of the variables when not involved in the interaction (clutch size and tarsus3) are irrelevant as in the case of an interaction, the effect of the variables involved should not be interpreted separately from the effect of the interaction

**(We have moved the majority of this paragraph to supplementary material, as this analysis is added to support our estimate of start weights. In the text, we state that we use a model with clutch size and tarsus3. Therefore, we believe that it should be clear that we only used one model and no model selection had been applied. We have presented test results for all variables in the one model we used, not just the significant variable.)**

L157: “in which hatching […] was recorded” **(done)**

L158: please add that Wstart is the response variable for clarity **(It is now stated clearly that w_hatch_ is the response variable!)**

L165-169: it would be informative to have the number of clutches of each size somewhere (could be in supplementary), to have an idea of the sample sizes of each clutch. **(This is a good idea. We have included a table in the supplementary material with the number of clutches of each size (S2 Table))**

L172-182: **1)** this paragraph is not very clear: does the first sentence refer to one analysis (LMM), then L174-182 to another one (ANOVAs)? **2)** Do the authors mean they ran repeated measures ANOVAs as post-hoc tests to the linear mixed model? Does individual nest (L178) refer to each egg within a same clutch or an actual nest (and then if a same nest is used several years is it considered as a different nest each year). **(Answer 1) The statistics paragraph has been rearranged and it should now be clear that the first sentence refer to a separate analysis. The sub headings in both sections should also erase the possibility for these distinctions between tests not to be clear. Answer 2) Good point as this was not clear. No. The repeated measures ANOVA (i.e. what used to be L.174-182) are a separate set of analyses from the analysis mentioned in what used to be L 172-173. The repeated measures analysis tested if egg size differed over the laying sequence between clutch sizes. Individual nest was actually the individual clutch.)**

L183: “their reproductive strategy” **(done)**

L183-191: this paragraph needs to be reformulated for more clarity, it seems that some sentences repeat the same information, and the type of analysis ran should be stated earlier. **(Some changes have been made to make reading easier. However, we do not find much repetition, only the “increasing, maintaining or reducing” is stated twice, being the response variable, and crucial for the understanding of how the analyses is structured. Furthermore, we have reformulated the part about the post hoc pairwise analysis.)**

L184: it is not clear if a season correspond to a year, or if inter-seasonal changes correspond to yearly changes **(changed to ‘year’ and ‘between year’)**

L188: it is unclear if individual refer to female ID. Please make sure to distinguish the significance of words that are used for different purpose (individual nest L178) **(change made to state that it is individual females)**

L191: please be consistent with the terminology used: egg size or egg volume (valid throughout the manuscript) **(done)**

L205: why present the R² here but not when presenting results from other models? **(We present R^2^ as an information on the negligible effect of year-to-year differences. R^2^ is not available for all types of models such as the generalized linear models.)**

L213: it is unclear what the authors mean by “shift in dominance”. Is it that in some years there were more 4-egg clutches while in other years 5-egg clutches were dominant? **(Yes, changes have been made)**

L218: there are a comma and a space missing between “0.0001” and “slope = 0.006”. Also please be consistent with “,” and “.” when writing numbers. **(done)**

L227-232: unclear: are females that lost weight more likely to reduce clutch size than females that gained weight, or than when they gain weight? Providing a figure would help represent this result **(The second sentence (L230-232) explicitly state how body mass changes relates to clutch size changes)**

L241-242: it is unclear if this refers to a difference between first eggs from different clutch sizes, second eggs from different clutch sizes, and third eggs from different clutch sizes, or if they refer to differences between first, second and third eggs across clutch sizes. Does it show the same results as Fig. 2 but in a different way? **(Changes made to make this point clear)**

L253-257: please state as a bottom note to the table that bold means significant effects and add sample sizes in the title. “data were analyzed with a mixed model” **(We have added sample size and added a note that bold numbers indicate significance.)**

L261-265: these sentences are a bit stating the obvious… Please reformulate to indicate the direction of the effects. **(Please note that both clutch size and order were coded as a discrete variable for which we cannot indicate a direction. We have now specified this in the methods, and we apologize for not making this clear in the first version. The reason for treating these as discrete variables was that it bypass the issue of linear or non-linear relations. Furthermore, the important result here is the significant interactions between order and clutch size, and here it is important not to obscure effects by forcing linear or polynomial relations upon them.)**

L267-271: please state as a bottom note to the table that bold means significant effects and add sample sizes in the title. “data were analyzed with a mixed model” **(We have included number of clutches in each of the analyses but note that this number is smaller than df for the error term as each clutch contribute with more than one observation in all tests except for the first test.)**

L275-277: please reformulate the sentence to make it clearer **(done)**

L279-280: please state the direction of the difference **(done)**

L281-282: please describe the relation documented **(with the corrections made to L279-280 it is clarified what this ‘opposite change’ is describing. Likewise, the following sentence state in more direct phrasing what these statistical test above actually means.)**

L307: “and the second egg marginally smaller than in 5-egg clutches.” **(done)**

L310-312: the indirect relationship between female body condition and within-clutch egg size variation does not suffice to conclude that egg-size variation patterns have an adaptive value. Plus, laying sequence has been suggested to be as strong a predictor of hatching and nestling survival as egg size. Without testing the effect of such patterns on a measure of fitness, within-clutch egg size variation as a reproductive tactic can at best be interpreted as a hypothetical explanation, hence should be discussed later in the discussion. **(We do not make any conclusion! As stated “our data suggest that…”. “is” is changed to “maybe” to avoid any misperception of this sentence)**

L325: is the environment really stochastic? **(word changed)**

L325: ‘affecting conditions for breeding females’ is unclear: do the authors refer to the impact of environment on female body condition? **(changes made)**

L331-333: please reformulate: the link between egg size and laying order has already been documented, as cited in the introduction (L50-54, L90-91). “for the first time” only applies to the link between within-clutch egg size variation, laying order and clutch size (second part of the sentence) **(changes made)**

L343-345: the larger first egg in 4-egg than in 5-egg clutches is significant only in ref 9 **(We only refer to “matching patterns” in other studies, not whether these patterns have been tested (significant or not significant). None of the three studies referred to makes specific analyses in relation to large first eggs in 4-egg clutches and small first eggs in 5-egg clutches, as this were not the aim.)**

L340-345: it is unclear what point this paragraph is making **(this paragraph highlight that the documented egg size pattern is not only found in the present study, but most probably is generally occurring, as these other three studies show the same pattern, even though they do not recognize it, or look for it, in their related studies. The reason that this pattern has not been recognized most probably relates to the relatively small difference in egg size involved, which we visualize here and that data over more years of the same female changing clutch size is very hard to get. We find this paragraph central in acknowledging that the within clutch egg-size variation found and analyzed in the present study is actually occurring in other eider populations.)**

L352: please remove the parentheses **(done)**

L363: “predator warning in females” **(done)**

L364-365: please reformulate, for example: “Such traits likely contribute to an increase in survival” **(done)**

L366-367: please rephrase this sentence to make it clearer **(changes made)**

L371: the formulation “in such a cohort ‘size-scenario’” is unclear **(changes made)**

L373-378: are the two percentage distributions statistically different, and statistically different from an expected one based on probabilities? (I’m just curious of the significance of these numbers) **(These numbers are not straight-forward to test, as they are just two numbers without an underlying distribution, at least the way that we see our data.)**

L380: please remove the parentheses **(done)**

L380-382: that is a really interesting point **(OK)**

L382: “abandon ducklings without impairing their survival” **(done)**

L387-390: please break the sentence down **(done)**

L405-406: good point, one potential measure of reproductive success that could be investigated in the present study to test the adaptive value of within-clutch egg size variation, since it was the aim of the study. **(As previously stated we do not have data to make such an analysis, hence it’s not our aim)**

L419-415: this paragraph repeats information given in L387-397. **(Only partly, and this section is related to the potential risk of introducing a developmental asynchrony in the embryos. Change have been made)**

L441-443: well, the study did not test that an increase in first egg size resulted in an increase in the number of large ducklings for 4-egg clutches. **(We do show that the proportion of large eggs/ducklings from 4 egg clutches constitute 47.6% of the largest ducklings in a cohort, which is larger than the estimated proportion (31.2%) if the size of the first egg in 4 egg clutches were following the more common pattern seen in all other clutch sizes!)**

L443-446: this is a hypothesis and not a result of this study, hence this sentence should be more nuanced. **(Changes made and “suggest” is swapped with “hypothesis”)**

L448-449: there is little information on the link between female body condition and environment in the manuscript, plus common eiders are capital breeders, hence this seems an odd conclusion to the paper. **(Changes made, including deletion of the word “environment”)**

Fig. 3: presenting these results following the same format as Fig. 2 would make comparisons easier to make for the reader **(Figure 2 describes the population sample whereas figure 3 and 4 describe individual changes. We therefore find it is ok that the figures differ in format. Y-axis legends has been changed from “egg volume” to “egg size)**

Reviewer #2:

The goal of this paper is to assess whether eiders adjust egg size within the laying sequence depending on clutch size, with the ultimate goal of understanding whether egg size adjustment is adaptive (via optimizing hatching size). This work utilizes an impressive dataset and provides comprehensive evidence that eiders lay first eggs are larger and second eggs smaller in clutches of 4 eggs compared to 5 eggs. I find the paper generally sound and most of my comments are minor and focus on clarifying potential points of confusion.

Line 35 and elsewhere - Rather than wording like “marginally insignificant”, it might be more clear to just say “showed x trend” or “tended to..” just to make the direction of the almost significant relationship more clear. **(Changes made)**

I found the estimates of female pre-laying body condition quite difficult to follow. Were individual females weighed both at capture and at hatching? Otherwise where does the annual mean mass at hatching come from? Although there are references to mass lost per day of incubation etc, there is no mention as to whether this formula was ad hoc or if there is a precedent for it. It seems to make sense but it takes the reader a while to understand so if possible I’d suggest a bit more detail as to how this particular formula was derived, as well as where the mean mass at hatching comes from. **(YES Only part of the females were weighed at hatching (with ducklings or piping eggs) and from regression of body mass on tarsus length of these, the average body mass for females at hatching was obtained which is clearly stated. With respect to the formula used, this is constructed for the present use, and is made as simple and descriptive as possible. Changes have been made in order to improve the readability.)**

The wording (particularly in the abstract) about this ‘adaptive’ strategy can come across a bit strong, given the way body condition etc were calculated and without a more comprehensive analysis of female energetics during egg laying/incubation and how it corresponds to increased survival of both the individual female and her chicks. I think the discussion is more moderate in tone so seems appropriate, but I think it would be prudent to adopt that language in the Abstract, rather than stating that this represents a “finely tuned conditional dependent mechanisms that enable females in a suboptimal condition to optimize reproductive output”, which seems a bit over-stated. **(Changes made to down-scale the wording of the final sentence)**
