# Supplementary material for: Condition dependent strategies of egg size variation in the Common Eider Somateria mollissima
Source: PLoS One. 2020 Jul 27;15(7):e0226532. doi: 10.1371/journal.pone.0226532 (PMC7384649; doi:10.1371/journal.pone.0226532)
Supplement: Attachment — Submitted filename: Response to reviewers.docx [file pone.0226532.s006.docx]

**PLOS ONE**

Online re-submission PONE-D-19-33049

*Review response*

Dear Editor!

On behalf of my co-author, Thorsten J. S. Balsby and I, I hereby submit the revised research paper entitled “**Condition dependent strategies of egg size variation in the Common Eider Somateria mollissima**”.

Please below in **red/bold** find a detailed response to all the excellent review comments and changes addressed. We have of course used all these excellent suggestions. All changes are shown in the highlighted version of our revised manuscript.

We are impressed by the reviewer’s excellent comments and suggestions, that definitely have improved the manuscript quality. We have included a personal thank to Pauline Toni in the acknowledgement of the manuscript, which we hope can be accepted by your journal; otherwise we will make this anonymous.

I am looking forward hearing from you.

Sincerely yours,

*Thomas Kjær Christensen
Senior researcher, PhD*
Institut for Bioscience, Aarhus Universitet
Grenåvej 14, 8410 Rønde, Danmark
Mail: [tk@bios.au.dk](mailto:tk@bios.au.dk) | tlf.: +45 87158854  cell: +45 40256813

6. Review Comments to the Author

Please use the space provided to explain your answers to the questions above. You may also include additional comments for the author, including concerns about dual publication, research ethics, or publication ethics. (Please upload your review as an attachment if it exceeds 20,000 characters)

Reviewer #1: This manuscript aims at testing how intra-clutch egg size varies with laying sequence depending on clutch size, and makes the link between such variation and maternal body condition, in common eiders. The authors use long-term, longitudinal data to answer these questions at both the population and the individual level. The study is of interest, as the results are sound and based on an impressively large dataset. The analyses at the population and individual levels bring important insights on egg size variation patterns. The paper offers an interesting discussion and interpretation of the results in the larger framework of female reproductive strategies.

This is the second time I review this manuscript (revised submission). I find that the authors have addressed appropriately the concerns that were previously raised, especially the reformulation of the study’s aims, the global organisation and the more modest tone used in the abstract. This revised version is much clearer, more precise, better organised and more readable than the previous one. I only have a few minor comments

The writing could still be improved, notably by shortening sentences, but the discourse is clear enough to be understood. Some English mistakes remain (I attempted at pointing them out in the specific comments).

Regarding the method section, great efforts have been made to provide more details on the analyses and the tests used, and to organise the analyses to mirror the results presentation. I think there is still one analysis description missing: I am guessing the results section entitled “Egg laying patterns within individuals” (L328-353) are from similar analyses to those described in the “intra-clutch egg-size variation” and “patterns of egg size variation in relation to laying order”, but this is not specified in the methods section. Adding one more subsection stating something like “we reproduced the analyses described in sections ‘…’, limiting them to recaptured females that had changed clutch size from 4 to 5 eggs between years” would do the job. Also, I think that, to ensure replicability and transparency, some more details could be provided, for example the models distribution when not using linear models (Poisson?).
**The statistics for the section: ‘ Egg-laying patterns within individuals’ was not described in the previous version. When examining it again we realized that our test did not account for the paired design. We therefore reanalyzed the data using a paired t-test. This did not change the significance of the results. We apologize for overlooking this issue earlier.**

As to the statistical features reported, I reckon it is a matter of opinion. I find slopes and SEs more informative as it provides with a direction and a strength of the differences. However, now that the statistics section is more detailed and much clearer than previously, I agree that F-values are inherent to the comparison process used in the analyses of this manuscript. As to the R² values, I have never used SAS, but is it not possible to manually calculate marginal and conditional R² for (G)LMMs, using Nakagawa and Schielzeth (2013 Methods in Ecology and Evolution) approximation? Just so that results are more consistently presented…
**We do not use R2 estimates for interpreting our results. We doubt that addition of R2 to the generalized linear mixed models would add much information. In addition, the R2 value is not simple to estimate in mixed and generalized mixed models, and the interpretation across statistical models is far from trivial.**

**Specific comments (line numbers referring to the manuscript version with tracked changes):**

Abstract
L22 and throughout: please harmonize between “intraclutch” and “intra-clutch” **Corrected**
L23: please add commas “to clutch size, and the relation between clutch size and female body condition,” **Corrected**
L25-26: I think the authors mean “within the laying sequence depending on clutch sizes in response to body condition” **Corrected**
L26: please reformulate “as such an adjustment could have adaptive implications on” **Corrected**
L28: “advantage for the hatchlings” **Corrected**
L28: “The analyses were first performed at the population level; and then at the individual level using data from” **Corrected**
L31-32: this part is a bit heavy, I suggest reformulating, for example as “(range: 1–6), 4- and 5-egg clutches constituted c.70% of all clutches, and taking turns in being the most represented clutch size.” **Corrected**
L34: “levels” **Corrected**
L39: if I understand correctly, the punctuation should be as follows “pre-laying body condition and clutch size, and the intraclutch” **Corrected**
L40: “pattern indicate that both clutch and egg size are actively” **Corrected**
L43: please nuance by replacing “indicates “with “suggests” **Corrected**
L44: “mechanism” (singular) **Corrected**
L45: “years where females are in suboptimal body condition” **Corrected**

Introduction
L61: “in a population” **Rephrased to “Different eider populations” (as this is shown in several different populations.**
L63: “of the first egg”; “efficiency of females” **Corrected**
L67-68: “number, to incubation strategy, to facilitation […] and to differential” **Corrected**
L72: “Although egg size” **Corrected**
L76: “which lay” **Corrected**
L83: by “equal”, do the authors mean “even” (as in ‘make equal’)? **Yes, new phrasing: “make equal”.**
L85: “life history” (no hyphen) **Corrected**
L93-94: “as well as an increase in reproductive success” **Corrected**
L102: “breeding populations, suggesting that eiders could” **Corrected, but the word “could” is not included, as this makes the sentence less precise.**
L105: “if common eider females change egg size” **Corrected**
L109-110: “data at the population level, as well as the individual level” **Corrected**

Methods
L120: “held” (not holded) **Corrected**
L133-134: “”in cases where no egg-shell remains were found to disqualify the record) **Corrected**
L156: do you mean “hatched eggs” (instead of hatching eggs)? **Hatching nests refer to nests where ducklings were not hatched, but where eggs were in the process of hatching. Hatched eggs is not a precise description as such nests will be similar to nests where females were captured with ducklings.**
L171: “To ensure” **Corrected**
L191: is there a difference between general linear model and generalized linear model (L203)? I suspect not, therefore please pick one. Also, if it is a GLM, please specify the response variable error distribution (I am assuming a Poisson one?) **General linear models assume normal distribution whereas generalized linear models assumes poisson or other non-normal distribution. We have specified the use of non-normal distributions (Generalized linear models) in the statistic paragraph.**
L208: do you mean “Intraclutch” (instead of Interclutch)? Cf. L279 **As these analyses consider both within and between clutch variation, we have changed the heading to “Inter- and intraclutch egg-size variation in both the Statistical and Result sections.**
L210: is it a linear mixed model? **As laying order was coded as a categorical variable and as such does not model a linear relation we think that mixed model is the most appropriate term.**
L211: “as a fixed effect and year as a random factor” **Corrected**
L215-216: “and year, and interactions between laying order and clutch size, and between year and laying order” **Corrected**
L218: “bypasses” **Corrected**
L220: “egg size could only be” **Corrected**
L221-222: “(e.g. the pattern for the first four eggs could only be tested in clutches” **Corrected**
L222-223: do you mean for each clutch size or that you conducted separate tests on first eggs, then on the first 2 eggs, then the first 3 eggs… (I assume the latter, therefore you might wann reformulate) **No. There needs to be at least 2 eggs in a clutch for testing laying order. We have rephrased the sentence.**
L238: “were fulfilled” **Corrected**

Results
L257: “with 4-egg clutches being more frequent in some years” **Corrected**
L261: “Clutch size was” **Corrected**
L263: “0.0001” (instead of 0,0001) **Corrected**
L266 & 268: “The majority of the recaptured females”; “size the following year” **First part is changed, but the second part (size the following year) is kept, as clutch size change did not in all instances take place from one year to the next. Some birds were not captured every year, but every second year.**
L267 & 269: remove “had” in both instances **Corrected**
L270: “Among the 108 females that did” (no comma) **Corrected**
L272: “and 65 gained” **Corrected**
L274-275: do you mean p < 0.0001 (instead of >)? No capital letter is needed for “slope” **Yes and** **Corrected**
L296: there is one too many closing parenthesis **Corrected**
L301: “data were analysed” (data is plural) **Corrected**
L314: please remove “as indicated by the significant effect of laying order” **Corrected**
L334: with “marked”, do you mean “marginal”? **No. With the t-test applied the significance is marked**
L338: please replace “evident” with “observed” **Corrected**

Discussion
L364-365: please remove “(although just insignificant)”. I understand you want to remind this point, so I suggest you replace “marginally” by “almost significantly” **Corrected**
L366: “females when they both increased” **Corrected**
L368: please replace “data” with “results” **Corrected**
L378: “laying date varied yearly? by only” **We have changed “colony” with “annual” to point out that yearly laying dates vary by only 5 days**
L389: “reflect” (data is plural) **Corrected**
L394: “with” instead of “within”, as I assume you mean females produce eggs according to a specific pattern **Corrected**
L397: “contemporary” is not too clear, I would use “associated pre-laying” **Corrected**
L413: do you mean “in terms of number of ducklings”? YES- **Corrected**
L414: “duckling survival” to what? **We have specified this: “higher duckling survival is the referred consequences.**
L418: “in size with the two” **Corrected**
L422: “the likely associated fitness benefits.”; “waterfowls?” (I am not sure if it is a mistake or if fowl has an irregular plural, same L450) **First part corrected. Second part: new phrasing is “waterfowl species”, which should be the correct (changed both places).**
 L433: “Analysed in relation to” **Corrected**
L435: “show” (data is plural) **Corrected**
L441: “5- and 6-egg” (there are some hyphens missing every now and then, also L475, 493, 495) **Corrected**
L442: “in terms of simple size” is not clear, if you mean ‘regarding size’, I think that can be removed as the point is made by the rest of the sentence **Corrected** **- deleted**
L443: please remove “both”, or reformulate as it is unclear which two elements it refers to **Corrected**
L454: either ‘the highest energy investment” or “higher energy investment” (although I think you mean allocation, as you refer to an increase in egg size but not necessarily associated with a greater cost to the female?) **First part corrected. It should be clear that it is an “allocation” from the previous sentence.**
L456-457: “eggs is much higher” (refers to pre-incubation failure, singular) and “eggs is attributed” (refers to survival, singular) **Corrected**
L458: “start” (plural, refers to both nest attendance and incubation initiation) **Corrected**
L502: please replace “specific” with “recaptured” **Corrected**
L503: “when in poor condition” **Corrected**
L504: “we argue that” (no comma) **Corrected**
L508: “clutches, we hypothesise” **Corrected**
